# Ocean sprawl facilitates dispersal and connectivity of protected species

**DOI:** 10.1038/s41598-018-29575-4

**Published:** 2018-08-16

**Authors:** Lea-Anne Henry, Claudia G. Mayorga-Adame, Alan D. Fox, Jeff A. Polton, Joseph S. Ferris, Faron McLellan, Chris McCabe, Tina Kutti, J. Murray Roberts

**Affiliations:** 1 School of GeoSciences, Grant Institute, James Hutton Road, King’s Buildings, University of Edinburgh, Edinburgh, EH9 3FE United Kingdom; 2 National Oceanography Centre, Joseph Proudman Building, 6 Brownlow Street, Liverpool, L3 5DA United Kingdom; 3 BMT Cordah, Broadfold House, Broadfold Road, Bridge of Don, Aberdeen, AB23 8EE United Kingdom; 4 Institute of Marine Research, Bergen, 5005 Norway; 5 Center for Marine Science, University of North Carolina Wilmington, 601 S. College Road, Wilmington, North Carolina 28403-5928 United States of America; 6 Present Address: ECAP Consultancy Group, Spean Bridge, Argyll, PH34 4EG United Kingdom; 7 Present Address: KIMO, Aberdeenshire Council, Woodhill House, Westburn Road, Aberdeen, AB16 5HG United Kingdom; 8 Present Address: Joint Nature Conservation Committee, Inverdee House, Baxter House, Aberdeen, AB11 9QA United Kingdom

## Abstract

Highly connected networks generally improve resilience in complex systems. We present a novel application of this paradigm and investigated the potential for anthropogenic structures in the ocean to enhance connectivity of a protected species threatened by human pressures and climate change. Biophysical dispersal models of a protected coral species simulated potential connectivity between oil and gas installations across the North Sea but also metapopulation outcomes for naturally occurring corals downstream. Network analyses illustrated how just a single generation of virtual larvae released from these installations could create a highly connected anthropogenic system, with larvae becoming competent to settle over a range of natural deep-sea, shelf and fjord coral ecosystems including a marine protected area. These results provide the first study showing that a system of anthropogenic structures can have international conservation significance by creating ecologically connected networks and by acting as stepping stones for cross-border interconnection to natural populations.

## Introduction

The increasing spread of anthropogenic structures in the world’s oceans adds a significant new component to marine ecosystems. These come in diverse forms, from harbours and jetties constructed since antiquity, to offshore platforms and pipelines, and more recently, renewable energy devices. These create an offshore “ocean sprawl” that can negatively impact marine ecosystems in many ways, e.g., sound, pollution, disturbance, invasive species, habitat alterations^1,2^.

Besides the challenges, ocean sprawl presents novel conservation opportunities^1^ such as expanding geographical ranges^3^, increasing secondary production for fisheries^4^, or providing foraging sites for species whose habitats have been degraded^5^. Benefits may also include increased population connectivity. There is a lot more understanding about how connectivity is enhanced in non-native or invasive species over long distances due to ocean sprawl^1,2^. For example, the invasion of two azooxanthellate corals, *Tubastraea coccinea* and *Tubastraea tagusensis*, off Brazil, which had fouled oil platforms and which today have expanded their range by more than 3 500 km. In contrast to invasive species research, large-scale studies are missing on how anthropogenic structures influence the movement of protected or threatened species^6^. Yet, it is the outcomes of such movements that ultimately determine population dynamics, persistence, species’ distributions, and ecosystem function^7–9^. The conservation significance of anthropogenic structures in urban landscapes was recognised nearly a decade ago^10^: the potential for ocean sprawl to positively benefit ocean resilience calls for a similar longer-term and larger scale view of the seascape in which offshore installations are considered alongside natural ecosystems^1^.

In complex systems, resilience depends on the arrangement of nodes in a network including the density of node connections, the extent that individual nodes dominate in terms of connections, and the direction of these connections between nodes^11^. Analysis of dispersal pathways and network topology can reveal new information about population connectivity and resilience, including larval sources and pathways to help underpin marine management and policies from the coast to the deep open ocean^12–15^.

The present study adopts a network approach to consider the role that oil and gas installations in the ocean could play in species conservation and enhancing resilience. Individual offshore structures can, for example, help support protected species. For example, platforms in the Persian Gulf region attract large numbers of the endangered whale shark *Rhinocodon typus*, which aggregate in the area to feed on the spawn of fish that were attracted themselves by smaller prey around the platform^16^. Protected deep-sea corals such as *Lophelia pertusa* colonise the legs of oil and gas platforms in the Gulf of Mexico and North Atlantic^17–19^, underpinning the notion that disposal of platforms at sea (the “rigs-to-reefs” concept) could benefit protected corals by increasing population connectivity across a seascape fragmented by demersal fisheries^20^.

Despite these positive local effects on individual animals, the lack of larger scale studies on impacts of anthropogenic structures on marine connectivity^6^ needs to be redressed, especially for protected and threatened species. For sessile benthic species, individual-based Lagrangian particle tracking models (IBMs) coupled to ocean circulation models are the most feasible method to study connectivity. IBMs have greatly extended the capacity to predict larval trajectories across large areas of the open ocean^21^, estimate connectivity between habitats^22–24^ including populations of protected corals^25–27^, and have been used to help design and evaluate the effectiveness of marine protected areas (MPAs)^14,28,29^. IBMs can resolve time varying 3-dimensional dispersion of planktonic larvae over large spatial scales with high spatiotemporal resolution^30^. IBMs using realistic larval ecology are key: marine larvae do not generally behave passively^31–34^, and their behaviours, particularly depth preferences and vertical swimming abilities, have large effects on dispersal^35–37^.

Here, the North Sea basin is used to illustrate the potential for widespread dispersal between anthropogenic structures and from populations on structures to natural ecosystems. Thorpe’s^38^ original model of connectivity in the North Sea based on tidal, mean and dispersive flows suggested that structures in the northern geographical sector were unlikely to be highly connected. Today, the availability of high-resolution shelf-seas IBMs integrated with biologically realistic particles offers an opportunity to explore this further. The North Sea is a global hotspot of multiple stressors^39^ with a long history of oil and gas production since the late 1950s^40^ and more than 1500 oil and gas installations in the region. Protected species of corals colonise many of these^17,41,42^, therefore, North Sea oil and gas platforms could now be playing a significant role in the metapopulation dynamics and conservation of this protected species. Many natural cold-water coral populations off bordering nations such as Norway and Sweden have been severely degraded by historical fisheries exploitation and slow to recover^43^. In the Gulf of Mexico, corals on platforms are thought to help reduce localised extinction risk and stabilise naturally occurring populations because their ranges have expanded, and because platform corals may act as refuges for small populations that could act as sources of recruits in the event of mass mortalities^44,45^. Rapid shoaling of the aragonite saturation horizon^46,47^ also means that “shallow” cold-water coral populations are likely to become refugia for deeper populations, which will be the first exposed to corrosive waters in the coming century^48^. Species connectivity patterns that track climate change must be considered in the design of robust MPA networks^49^, but the spatial connectivity and potential sensitivity to climate change must first be determined.

In this study, we used a Lagrangian particle tracking algorithm including larval behaviour specific to the cold-water coral *L*. *pertusa* coupled to a high resolution ocean circulation model, and analysed the modelled connectivity using network theory metrics to investigate network variability. These simulations allowed us to (1) predict the extent to which installations have themselves formed a connected network, and (2) examine whether larvae released from populations on the installations could potentially disperse to areas with naturally occurring populations and thus increase resilience of the regional metapopulation.

## Methods

### Study area and selection of oil and gas installations

**Figure 1:**
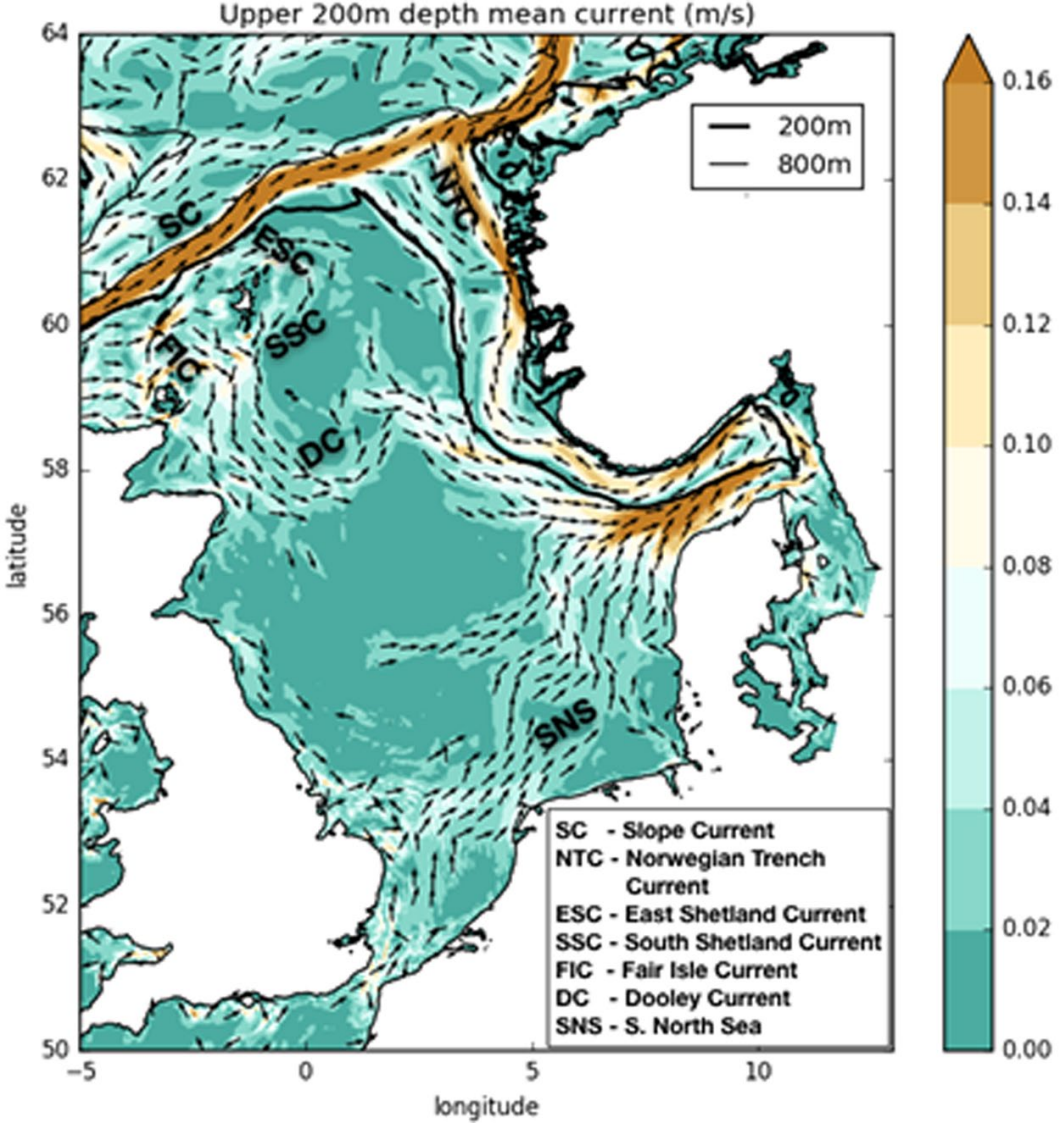
North Sea circulation computed from 2010–2012 time- and depth-average upper 200 m velocity field. Computed from AMM60 (Guihou, 2017). The 200 m and 800 m isobaths are contoured in black.

The study area was constrained to the greater North Sea region (Fig. 1). The installations extended from the shallow southern North Sea to the deeper northern North Sea. The depth increases from the English Channel and southern North Sea (~30 m depth) to the boundary with the Atlantic Ocean (~200 m depth) in the northern North Sea. The main inflow to the North Sea consists of Atlantic water following the 200 m depth contour north of Shetland before passing along the western edge of the Norwegian Trench (Fig. 1) with a smaller flow, the Fair Isle Current, following the 100 m depth contour (Fig. 1). The northern North Sea stratifies in the summer and can seasonally inhibit vertical mixing^50^. The circulation pattern on the North Sea ‘plateau’ is approximately equally influenced by wind, open ocean forcing and density, with tides playing an integral role in controlling the stratification^51^. The resulting circulation generally flows along isobaths and is enhanced over steep bathymetry, i.e. along the Atlantic and Norwegian Trench margins, resulting in a balance between fresh Baltic outflow and saline Atlantic waters^52^ (Fig. 1).

The OSPAR Inventory of Offshore Installations (data retrieved from www. https://odims.ospar.org/documents/686; last accessed 24 October, 2017) was used to generate an initial list of nodes from this region. Underwater video surveys conducted by remotely operated vehicles (ROVs) from 11 different oil and gas structures in the North Sea were reviewed. Depth ranges of *L*. *pertusa* and depth of peak coral cover were recorded to obtain a mean depth range to seed the larvae from. Using information from the marine growth survey analyses (SI Table S1), a final list of structures thought to have been colonised by *Lophelia pertusa* was selected to run the experiments. This was based on whether the mean depth range of *L*. *pertusa* from the marine growth surveys was deeper than the water depth of the structure, i.e., structures shallower than 80 m water depth were not used in the experiments (SI Table S1).

### Individual-based Lagrangian modelling (IBM) experiments

Particle tracking experiments were performed using the Lagrangian TRANSport model (LTRANSv.2b^53^) modified to work with outputs of a high resolution configuration of the Nucleus for European Modelling of the Ocean (NEMO) for the North West European Shelf, the NEMO-AMM60 (Atlantic Margin Model 1/60°^54^). This model has a horizontal resolution of approximately 1.8 km and 51 stretched-σ vertical levels with realistic GEBCO bathymetry. It includes atmospheric (ERA-interim), tidal (TPXO7.2) and open boundary (ORCA0083) forcings, as well as climatological river inputs^54^, providing realistic 3-dimensional current velocities. 1-hourly velocity fields were used to accurately represent the strong tidal flows characteristic of the North Sea. Particles were advected in the horizontal and vertical directions by the model velocities. A vertical random walk, scaled by the 3-dimensional spatially variable vertical viscosity coefficient of the model, was added to the particle’s motion to mimic diffusivity at sub-grid scales.

Virtual larvae were configured within LTRANS to mimic ontogenetic changes in larval behaviour, with swimming speeds and direction of motion varying depending upon age^55,56^. *Lophelia pertusa* is gonochoristic and spawns between January and February in northern Europe^55,56^. Larval swimming speed increased linearly with age from 0 to 0.5 mm s^−1^ between age 0 and 14 days. After 14 days of age, virtual larvae swam at the constant maximum speed of 0.5 mm s^−1^. Direction of particle motion was assigned by a weighted random component to reflect ontogenetic changes. A particle was assigned a random number between 0 and 1: if the number was less than or equal to the weighted value of 0.8, then the particle travelled towards the preferred direction, and if it was >0.8, it travelled the other way. Thus, at any given point in time, 80% of the particles would travel in the preferred direction. In this way, particles would have a tendency to move up and remain in the upper 20 m of the water column until they reached competency at 32 days of age. After this, they became bottom-oriented with a tendency to stay within 1 m of the bottom.

Every day at midnight 2,185 particles representing packets of larvae (super-individuals) were released for 14 days (days 15–28) in each spawning month (January and February) at 5 m depth intervals over the mean depth range (80–113 m water depth) based on the industry marine growth surveys, for a total of 61,180 super-individuals released per year. Releasing particles nightly over 14 days means they will be released over a full range of the tidal semi-diurnal cycle, a spring neap cycle, and will also sample the non-tidal background flows. This design (in particular sampling across the tidal cycle) ensures the particle paths are representative of all likely trajectories. Particles were tracked for 60 days with a time-step of integration of 10 minutes and their along-track locations stored every 30 minutes. Virtual larvae were assumed to become competent to settle at 32 days of age and remain suitable for settlement until the end of the simulated pelagic larval duration. A lose enveloping polygon was drawn around the cloud of positions occupied during competency by all particles originated from each subsea structure (See SI Figure S1). These polygons were assumed to represent the footprint during competency of each platform, and were devised as a way to compensate for sub-grid scale horizontal diffusion and the number of particles released, which was limited by computer resources. Subsea structures were considered to be connected if they lay within the enveloping competency polygon of another structure. This method is superior to the classical method in evaluating settlement locations for non-divergent flows since it “fills holes” that would not exist if sufficiently many particles were used (see SI Figure S1). The polygon method only works if the particles released sufficiently sample the possible spread in dispersal pathways. A conservative “strength of the connection” metric was diagnosed in two stages as the minimum number of connections made between two installations. Firstly, if a connection was found using the polygon method, then it was attributed a minimum connection strength of 1. Secondly, if a connection was found using the classical trajectory-following method, then the connection strength was substituted by that number. In the classical method, connections were counted directly as particles pass within a settling radius of 1 km of an installation (where 1 km is less than, or the scale of, the tidal excursion^57^). Settlement was evaluated every 30 minutes.

### Ocean state scenarios

Particle release experiments were performed in years 2010, 2011 and 2012 in order to account for the strong link between the North Atlantic Oscillation (NAO) and coral larval dispersal. In western Europe, sub-decadal variability in large-scale air-sea interactions creates different annual states of the NAO^58^ that could affect network connectivity by altering the strength and direction of westerly winds and the inflow of Atlantic waters^26^. A conservative “base-case” scenario of connectivity between installations was run for the year 2010 when westerly winds and inflow from the Atlantic are assumed to have been supressed^26^ by the negative NAO conditions. Subsequent simulations were run for 2011 and 2012 in a run up to a strong positive NAO year when it was assumed that westerly winds and Atlantic inflow would be greatest into the North Sea. This set-up allowed us to assess inter-annual variability in connectivity and larval trajectories.

### Network analyses

**Figure 2:**
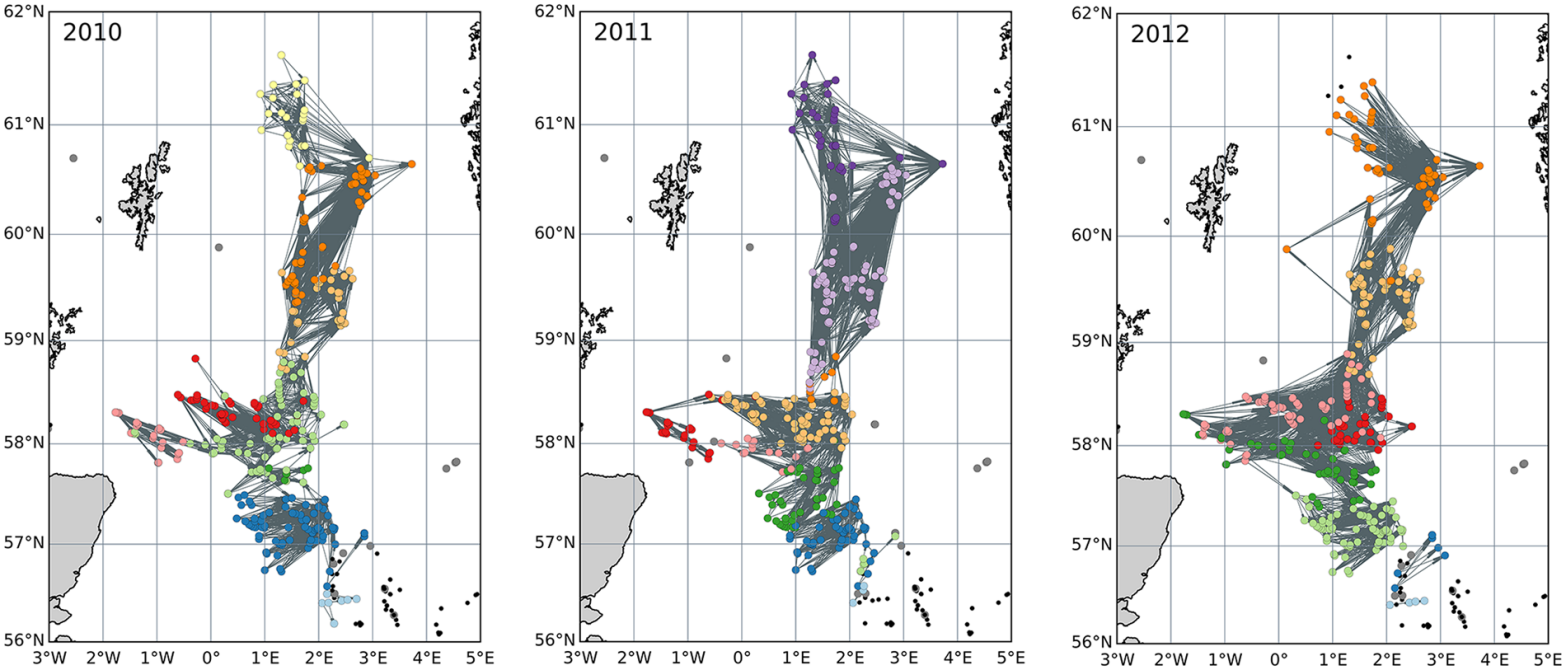
Inter-annual variability in network topology for the protected coral *Lophelia pertusa*. Highly connected clusters of installations are colour-coded based on Louvain modularity. Grey lines indicate structures that are connected. Grey dots indicate structures likely to have *L*. *pertusa* present; black dots represent structures unlikely to have *L*. *pertusa* present and thus excluded from all analyses.

Connections between installations for each year were visualised to illustrate network topology including clustering and direction of larval flow between structures (Fig. 2). We applied Louvain modularity^59^ to measure how strongly a graph was divided into clusters compared to a random graph using the python community detection for NetworkX package. Louvain modularity uses a resolution parameter^60^ to determine the number of clusters selected: our networks appeared relatively insensitive to the choice of resolution parameter, so we used a default value of 1.0.

Various network analysis metrics were calculated for each year: (1) *In-degree* and (2) *out-degree*. These were calculated both as unweighted counts of the number of connections, and weighted counts of the numbers of connections, including self-loops (i.e. returning larvae seeded from the same installation), into and out of each site. Unweighted and weighted degree metrics, although measuring different quantities (number of sites linked to/from and number of connections supplied/received respectively) show very similar spatial patterns. We focus on unweighted degree, with weighted degree included as Supplementary Information; (3) *Larval retention*. The number of particles retained at each site was calculated. These were found to be small compared to total numbers of particles settling (typically <2%) and is included in Supplementary information; (4) *eigenvector centrality* measures the importance of an installation, based on the idea that an important site is one that supplies virtual larvae to (so-called right eigenvector) or receives larvae from (left-eigenvector) other important sites. Here, we present the *right eigenvector centrality*, for the identification of major sources of virtual larvae, the complementary left eigenvector centrality was found to be less variable and informative and is included in the Supplementary Information. Eigenvector centrality was calculated using the weighted adjacency matrix with weights *w* normalised to 0 < *w* < 1, the higher weighting corresponding to a stronger connection; (5) high *betweenness centrality* identifies installations which lie on the shortest path between many pairs of sites, such sites may act as important bridges or stepping-stones, holding the network together. The path length, or distance, between two sites can be defined as a function of the connection weight^61^:

$${d}_{ij}=\,\mathrm{ln}(1/{w}_{ij})$$

where *d*_*ij*_ is the distance from node i to node j and *w*_*ij*_ is the strength of the connection. Note that stronger connections correspond to shorter distances.

Variability in all metrics was visually inspected across all three years to determine how robust these metrics were to inter-annual changes in climate and ocean circulation.

### Connectivity to natural populations

Particle trajectories for each year were plotted using python and the matplotlib package. In order to investigate the overlap between larvae dispersed from anthropogenic structures and natural populations, trajectories were mapped onto additional layers of geospatial data describing the distribution of areas with high densities of coral and locations of marine conservation areas in Norway’s Exclusive Economic Zone (EEZ). Data from the MAREANO project (www.mareano.no/en), which mapped the seafloor, biodiversity and habitats in the offshore region of Norway, were used as overlays with the new particle trajectories.

## Results

Integrated NEMO-AMM60-LTRANS particle tracking modelling, network analyses and *in situ* observations illustrated the strong potential for oil and gas installations to have significant conservation significance to protected species. Installations had the potential to form a highly inter-connected network of coral ecosystems that can enhance ecosystem resilience of natural populations.

### Platform inter-connectivity

#### Network topology

High inter-connectivity was a robust feature across different NAO states with between 8–11 smaller clusters of installations identified within the wider network (Fig. 2). The boundaries of these clusters were largely zonal and consistent across the three years. Evolution of connectivity in time in larval direction broadly conformed to an increase in zonal connections from west to east, and a reduction of meridional connections between/from 2010 and/to 2012 (Fig. 2). Installations located between 0–2°W became connected to the network by 2012. The installation furthest west (and closest to Scotland’s mainland) went from supplying virtual larvae to only one platform in 2010 and 2011, to supplying virtual larvae to five platforms in 2012 (Fig. 2), and the increase in connections to installations located between approximately 2–4°E off western Norway are examples of increased zonal connectivity in 2012 (Fig. 2). Only the most northerly installations lost connectivity over the time period 2010 to 2012, for example, note the loss of connections north of 61°N (Fig. 2).

#### In- and out-degree

**Figure 3:**
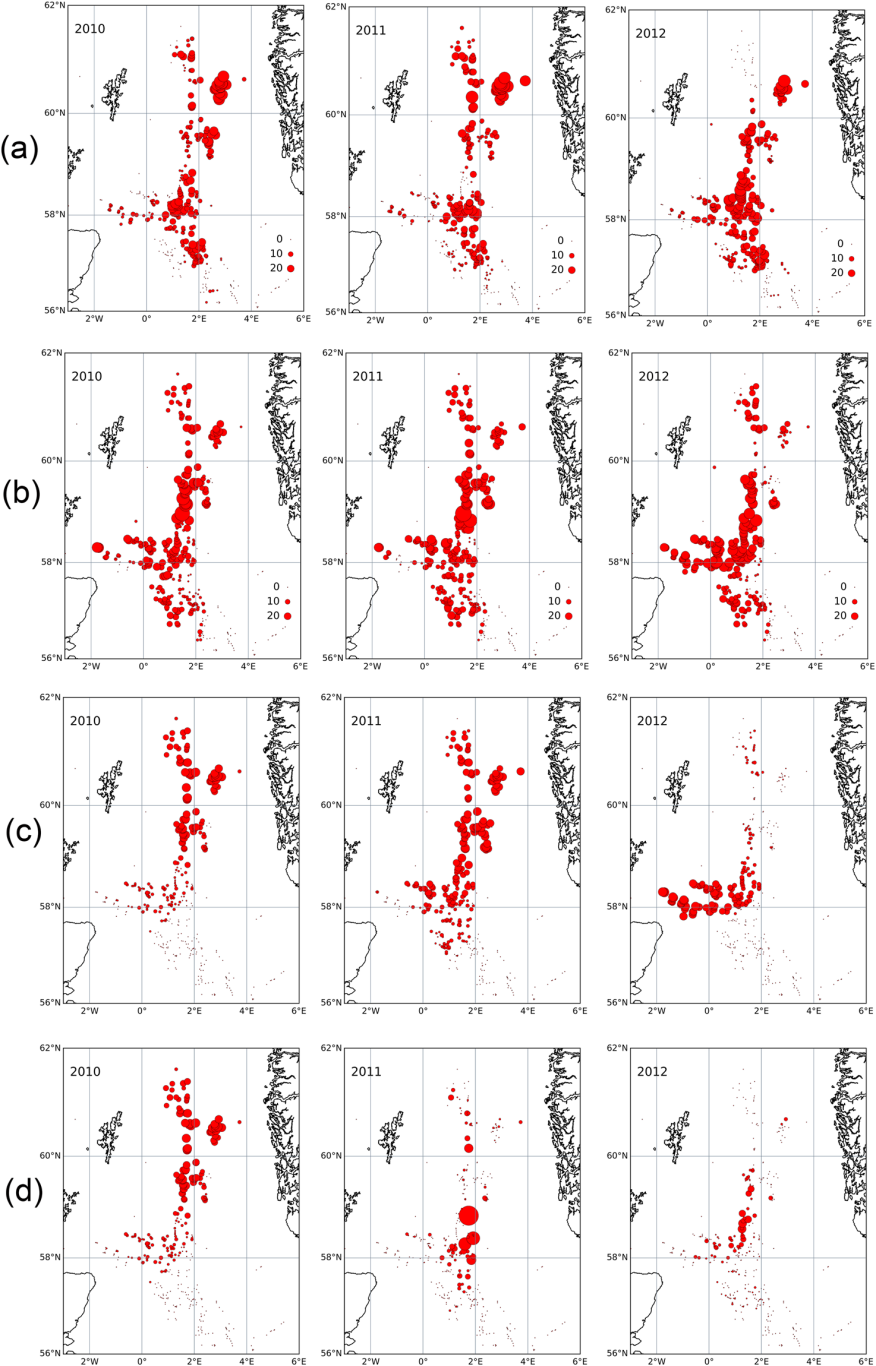
Network metrics including (**a**) in-degree, (**b**) out-degree, (**c**) log-scaled eigenvector centrality, and (**d**) betweenness for the putative system of *Lophelia pertusa* populations on oil and gas installations in the North Sea spanning a strong negative (2010) to positive NAO state (2012). Circle size indicates the magnitude of the metric. For simplicity, structures that do not connect to any other structures are not shown.

Plots of in-degree showed a lot of spatial heterogeneity (Fig. 3a, SI Figure S2a). However, in-degree became more uniformly distributed across installations by 2012 (Fig. 3a) when the network topology could be seen to have become more connected as west to east transport had increased (Fig. 2).

Spatial trends in out-degree were less pronounced than in-degree, with the same clusters of installations generally supplying similar numbers of larvae to other structures over the 2010–2012 time period (Fig. 3b, SI Figure S2b). There was a weak but increasing trend in the southernmost installations and a single northern installation supplying more larvae by 2012 (Fig. 3b), but on the whole, out-degree became even more uniform across clusters as west to east transport increased (Fig. 2).

Self-seeding (SI Figure S2c) was always small (<2% of total modelled larval settlement) although it was larger in the weaker mean flows of 2010 than in 2011 or 2012). Larval return, either direct retention or via a number of sites and generations, represents a crucial element for the persistence of metapopulations (Hastings and Botsford, 2006). Modelled connectivity may be underestimating larval retention through not resolving the fine scale turbulent processes around the structures.

#### Eigenvector centrality

Left eigenvector centrality, the extent to which installations were connected to other highly connected structures, varied strongly across years. This variability trend manifested itself as relative increases in eigenvector centrality in the southernmost installations around 58°N, with these structures becoming highly connected by 2012 relative to structures further north where any signal of high centrality had dissipated (Fig. 3c). In 2010, the most important source region was around 59–61°N, while in 2012 it was strongly concentrated around 58°N. In 2011, the important sources are more widely distributed.

Right eigenvector centrality (SI Figure S2d) showed little variation in structure, being dominated in all three years by downstream sites around 3°E, 60.5°N.

#### Betweenness

The relative importance of installations as bridges linking the different clusters (betweenness) strongly varied across years although sites around 58.5–59°N generally had relatively high betweenness. This latitude forms a ‘pinch point’ in the network topology with relatively few platforms all concentrated in a narrow zonal band forming a north-south link. Betweenness was highest in 2010, when the more southeasterly installations dominated as important bridges (Fig. 3d). These sites sit between the important source region further north (Fig. 3c) and sites in the highly connected cluster further to the south and west (pale green sites in Fig. 2a). There is not a straightforward relationship between the absolute values of betweenness each year and network connectivity: both stronger, longer connections and lost connections can lead to an overall drop in betweenness centrality. However, in 2011 and 2012 during the positive NAO spin-up, installations generally became more connected (Fig. 2) over longer distances from west to east, making the network less dependent on installations that could bridge otherwise disconnected clusters. A corresponding reduction in east-west connections in the stronger eastward flows may also act to reduce maximum betweenness in 2011 and 2012.

### Larval trajectories in relation to naturally occurring coral ecosystems

**Figure 4:**
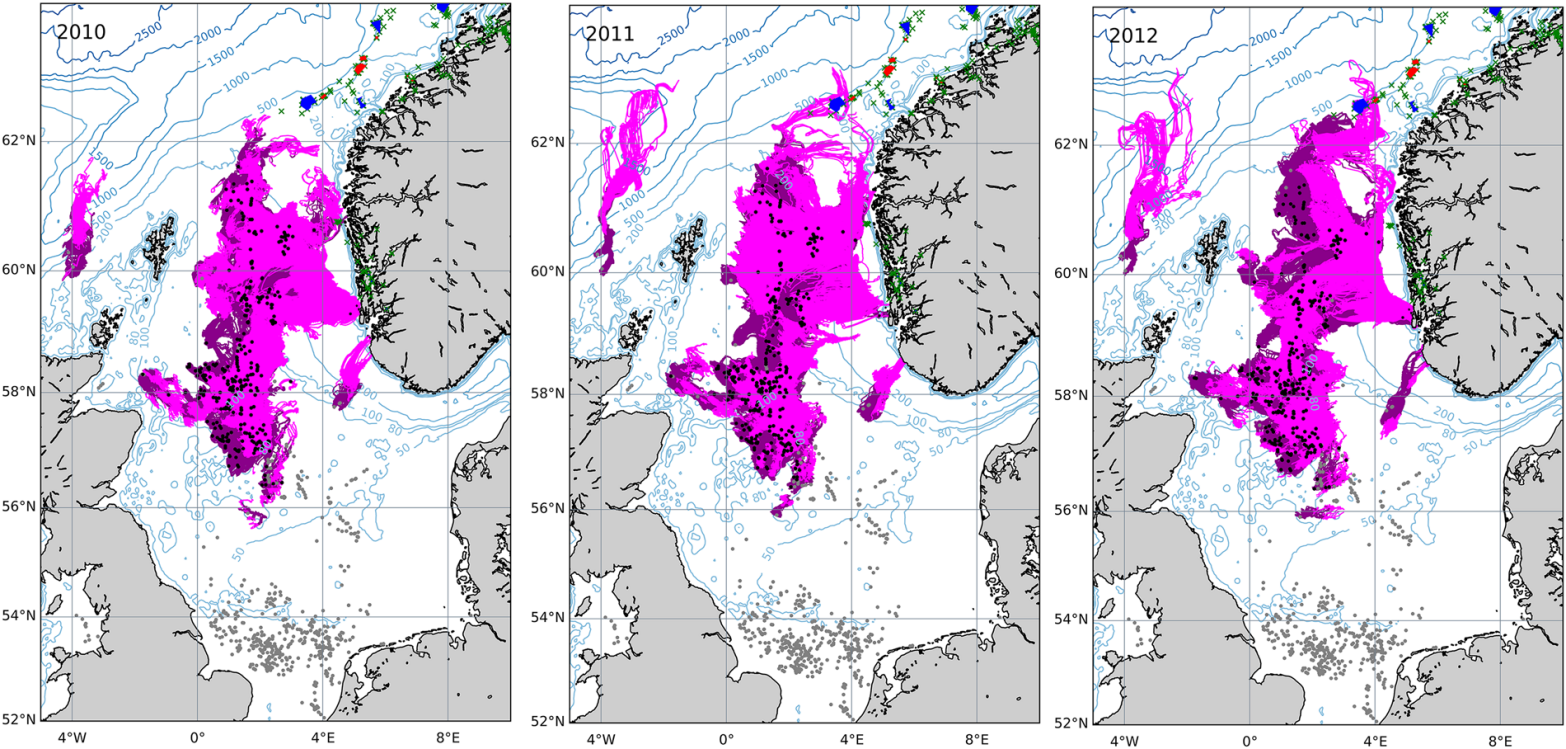
Inter-annual variability in dispersal pathways of coral larvae released from North Sea oil and gas structures. Bathymetric depth contours in metres illustrate pathways of pre-competent (dark pink) and competent (light pink) larvae over deep-sea, shelf and fjordic coral ecosystems. Blue and red polygons indicate coral marine protected areas and zones of high coral density, respectively. Black (grey) dots indicate oil and gas structures included (excluded) from the analyses. Green crosses indicate occurrences of natural populations off the coast of Norway.

Particle trajectories illustrated the potential for larvae to reach distant geographic locations in the North Sea and beyond, crossing international borders from Great Britain to Norway. Simulated larvae became competent to settle over large geographical areas, particularly in 2012 (Fig. 4) when larvae reached a variety of natural coral ecosystems including those in the deep sea, the continental shelf and in fjords (Fig. 4). Exceptionally, in 2012, larvae seeded from two platforms (*Murchison* and *Thistle Alpha*) became competent to settle over Norway’s Aktivneset coral marine protected area (SI Figure S3). Ground-truthed observations of *Lophelia pertusa* colonising *Murchison* and *Thistle Alpha* were confirmed by inspection of unpublished industry ROV video and previous publications^18,62–64^.

## Discussion

The present study offers the provocative suggestion that ocean infrastructure can have large-scale conservation significance to protected species. Simulations illustrated how North Sea oil and gas installations have the strong potential to form highly inter-connected regional network of anthropogenic coral ecosystems capable of supplying larvae to natural populations downstream. The long history of exploitation of resources in the North Sea has resulted in a multitude of hard substrata introduced into the wider ecosystem, including not only oil and gas platforms and wells, but also wrecks, marine renewable structures, pipelines, piers and shorebreaks. The new results from the present study allow us to consider the role that these “outlier” unnatural ecosystems can have for species resilience and conservation, especially if they have now formed their own inter-connected network of populations and ecosystems.

### The North Sea’s dynamic network of coral ecosystems

Our simulations of virtual larvae showed that since the 1970’s, development of North Sea oil and gas installations has likely created a connected network of natural and anthropogenic coral ecosystems that today spans hundreds of kilometres crossing international borders (Fig. 2). Inter-annual variability in both climate state (NAO) combined with the industry’s life cycle of assets from installation to decommissioning and removal mean that the entire network must be viewed as spatially and temporally dynamic.

The method used for finding network connections is a conservative estimate of the total connections possible. The bounding polygon method closes gaps in the classical trajectory method but in itself is no guarantee that the fluid environment is appropriately sampled (which is why we release super-individuals to sample natural variability of the tides, at the semidiurnal and spring-neap frequencies, and to coarsely sample that NAO). So while we can interpolate connections to span the range of directional spread, we cannot extrapolate the connections to extend their range. Nevertheless, we have shown that the polygon method saturates, with respect to the number of new connections, more rapidly than the classical trajectory approach. In these experiments, computational resources are distributed evenly to discover connections between all installations such that network saturation was not computationally feasible (as it would be if a single source installation was investigated). Consequently the connections discovered in this study constitute a conservative estimate of the total possible connections.

Model predictions demonstrated that the most densely connected clusters of oil and gas installations were those located closest to one another (Fig. 2), as would be expected under the general paradigm of stronger genetic and ecological isolation with increasing geographic distances in the marine realm^65^. However, connectivity across larger distances and across clusters was also predicted, reflecting how dispersal-driven connectivity is mediated by a combination of intrinsic life history traits and extrinsic environmental factors^66,67^. The capacity for long-range dispersal also agrees with previous observations on Lophelia’s postglacial fossil coral history^67,68^, larval behaviours^55,56^, and population genetic structure^68^. These studies related *Lophelia*’s ability to rapidly colonise new areas across vast geographic distances to the dispersal potential of *Lophelia*’s planktonic free-swimming larval life stage and the postglacial resumption of strong ocean currents.

Cross-cluster connectivity was maintained by a limited number of installations acting as bridges (Fig. 3d). The southernmost platforms had the highest betweenness, with only sparse connections to other structures visible in the network topology (Fig. 2). Thus, under conditions of reduced Atlantic inflow, these installations would serve as bridges critical to maintaining the overall network. The relationship between the occurrence of bridging nodes and the NAO phase also reflects state-of-the-art understanding that such bridges, or “stepping stones”, become increasingly more important to connectivity as ocean currents weaken^69^. The role of anthropogenic stepping-stones is important to consider for adaptive spatial management plans because over the next century the Atlantic’s ocean conveyor belt, the Atlantic Meridional Overturning Circulation, is expected to slow^70^ and could push the NAO into record lows^71^ that would reduce connections (Fig. 2) and cut off larvae to natural coral ecosystems and MPAs (Fig. 4).

### Network control to enhance resilience

The new results illustrate the potential for ocean sprawl to create a new highly inter-connected system of its own, potentially capable of enhancing resilience across the meta-population and in populations degraded by human activities (Fig. 4). Resilience depends on the density of node connections, the heterogeneity and symmetry of the links coming into and originating from individual nodes^11^. The present study illustrated that in the real-world scenario of North Sea oil and gas installations, anthropogenic structures could have, over several decades, facilitated the creation of a system of densely inter-connected clusters of coral ecosystems, with both natural and artificial reefs (Fig. 2). However, connections between anthropogenic structures displayed asymmetry with larvae dispersing mostly in one direction, tracking predominant currents (Fig. 2). This directionality also meant that some structures had higher centrality than others (Fig. 3c), making connectivity heterogeneous over both space and time. Thus, resilience of this new anthropogenic network and any downstream populations they supply is vulnerable to changes in circulation patterns and removal of structures with the highest centrality, i.e., those in the southerly extent of *Lophelia*’s distribution in the North Sea.

### Conservation significance of platform corals

Larval trajectories and *in situ* industry data from *Thistle Alpha* and *Murchison* illustrated the potential for some structures to contribute to the wider marine meta-population and the regional ecology and biodiversity of the North Sea ecosystem. The Aktivneset coral MPA is located in Storegga on Norway’s steep western continental shelf, an area with extensive historic fisheries damage to coral ecosystems. Dense coral occurrences in Aktivneset were noted in waters 270–350 m deep, but ROV surveys showed that trawling damage had taken its toll on much of the area^72^. It was also remarked that the likelihood that such damage would reduce coral reproductive potential, but that *Lophelia*’s planktonic larvae may be capable of recolonising damaged reefs from distant undamaged locations^72^. Our study demonstrated the potential for platform corals to help seed larvae to large areas across Norway’s deep sea, its continental shelf and fjord ecosystems, including the Aktivneset MPA (Fig. 4, SI Figure S1). Notably, the *Murchison* platform ceased production in 2016, with the last 44 m of the jacket footings being left in place on the seafloor, thereby leaving corals located deeper than 112 m water depth intact and thus capable of continuing to produce larvae.

The potential for platform corals to supply degraded areas has already been noted in mature production basins such as the Gulf of Mexico^45^, and it could be concluded that in some cases, key sources of larvae for protected species such as some oil and gas platforms could be made part of a larger MPA network. However, the benefit of increased connectivity and metapopulation resilience must be considered alongside any legacy effects of the contaminated drill cuttings that have over 40 years accumulated into a large pile. The macrobenthic community around *Murchison* appears to have recovered from historic cuttings disturbance and contamination, but re-disturbance could reset this recovery trajectory^73^. Furthermore, contaminated cuttings could significantly reduce the true dispersal potential through direct mortality of larvae trying to settle in areas with high pollutant loads^74^.

The application of our IBMs was the first step-change in understanding how such structures can contribute to ecological connectivity and metapopulation resilience: the next step-change can be made through ground-truthed verification of the linkages^75,76^ and for these to be scaled by reproductive input^77^ and settlement^22^. This combined evidence should then be considered alongside the weight of a multitude of other environmental, technological and socioeconomic factors when making the case to remove or leave structures in place on the seafloor at the end of their operational lifetime^78^.

As with studies on seawall defense structures providing conservation benefits to key ecosystem engineers such as fucoid algae^79^, it is anticipated that further scientific effort in this domain will continue to identify new ways for marine infrastructure to enhance populations of threatened species^79^. It is important to note that even before the end of infrastructure operational lifetime, more consideration could also be given not only to the cumulative role that adding new offshore structures could have, e.g., enhancing marine connectivity in MPAs^80^, but also the technical design, specification and arrangement of structures that could further enhance the ecosystem towards a desired state or conservation target, e.g., Good Environmental Status under the European Marine Strategy Framework Directive^80^. It is here that the very well-developed knowledge base on artificial reefs and materials to enhance biodiversity needs to better integrated into the initial design and engineering phases. Decades of research on artificial reefs is now set to move the application of anthropogenic structures from fisheries enhancement to having an even wider set of goals including recovery and rehabilitation of marine ecosystems^81^. Collaborations among ocean stakeholders including those that hold data on ecosystems inhabiting structures will also be key to informing future planning decisions^82^.

## Electronic supplementary material

Supplementary Information
