# Supplementary material for: Ocean sprawl facilitates dispersal and connectivity of protected species
Source: Sci Rep. 2018 Aug 16;8:11346. doi: 10.1038/s41598-018-29575-4 (PMC6095900; doi:10.1038/s41598-018-29575-4)

Supplementary Information

Ocean sprawl facilitates dispersal and connectivity of protected species

*Lea-Anne Henry[1], Claudia G. Mayorga-Adame[2], Alan D. Fox[1], Jeff A. Polton[2], Joseph S. Ferris[3], Faron McLellan[3], Chris McCabe[3], Tina Kutti[4], J. Murray Roberts[1,5]

[1]School of GeoSciences, Grant Institute, James Hutton Road, King's Buildings, University of Edinburgh, Edinburgh, United Kingdom, EH9 3FE

[2]National Oceanography Centre, Joseph Proudman Building, 6 Brownlow Street, Liverpool, United Kingdom, L3 5DA

[3]BMT Cordah, Broadfold House, Broadfold Road, Bridge of Don, Aberdeen, United Kingdom, AB23 8EE

[4]Institute of Marine Research, Bergen, Norway, 5005

[5]Center for Marine Science, University of North Carolina Wilmington, 601 S. College Road, Wilmington, North Carolina, United States of America, 28403-5928

*corresponding author: +44(0)131 650 5425 (telephone); l.henry@ed.ac.uk (email)

SI Table S1: Depth ranges of the coral *Lophelia pertusa* recorded from industry ROV marine growth surveys.

**Structure**	**Year of survey**	**Maximum depth surveyed (m)**	**Minimum depth observed (m)**	**Maximum depth observed (m)**	**Depth with peak coral cover (m)**
Dunbar	2014	137	106	126	116
Alwyn A	2014	90	58	90	90
Alwyn B	2014	89	52	80	71
Ninian North	2011	141	55	133	133
Murchison	2009	87	59	87	87
Murchison conductor	2009	153	59	140	140
Ninian South	2007	138	58	138	74
anonymous	1999	100	100	100	100
Ninian North	1999	100	100	100	100
Murchison	2011	155	155	155	155
anonymous	2012	102	74	97	97
		**Mean**	**80**	**113**	**106**

SI Figure S1: Schematic illustrating how connections between installations (crosses) are found with settling radii (enclosing circles). Super-individual larvae are released from a source installation (cross, lower left). The bounding envelope of the paths is shown for three such particles (dotted lines). The competency period is shaded in yellow and two installations are settled on. A loose polygon is then drawn around all the locations visited by all the particles during the competency period (dashed line). The additional area (shaded blue) contains two more connections. A total of four connections are found in this example.

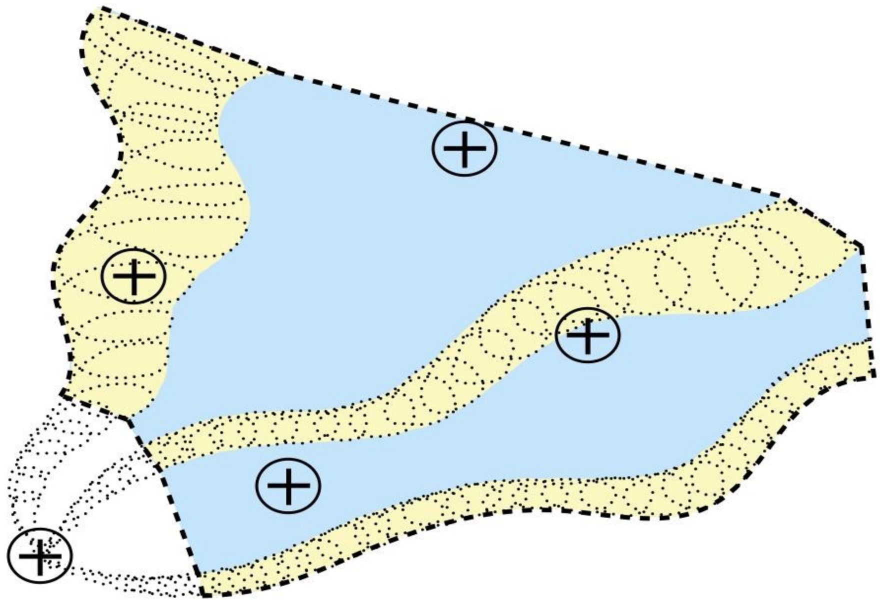

SI Figure S2: Network metrics including (a) weighted in-degree, (b) weighted out-degree, (c) larval retention, scaled up times 50 compared to in and out degree in (a) and (b), and (d) log-scaled right eigenvector centrality, for the putative system of *Lophelia pertusa* populations on oil and gas installations in the North Sea spanning a strong negative (2010) to positive NAO state (2012). Circle size indicates the magnitude of the metric.

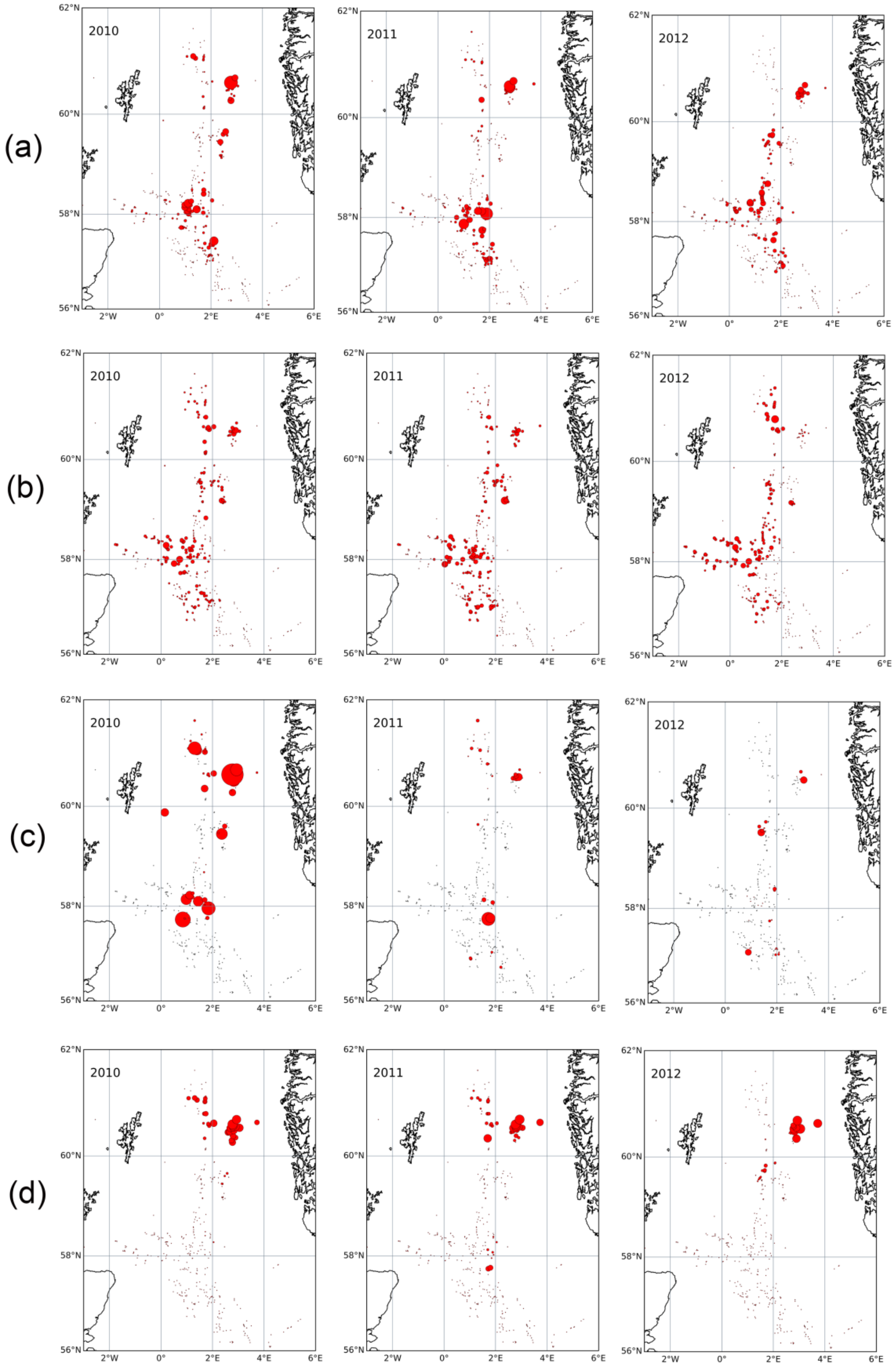

SI Figure S3: Particle trajectories of *Lophelia pertusa* larvae released from the Murchison (top) and Thistle Alpha (bottom) platforms in the North Sea. Pre-competent (dark pink) and competent (light pink) larvae disperse over deep-sea, shelf and fjordic coral ecosystems. Blue and red polygons indicate coral marine protected areas and zones of high coral density, respectively. Grey dots indicate oil and gas structures excluded from the analyses. Green crosses indicate occurrences of natural populations off the coast of Norway.

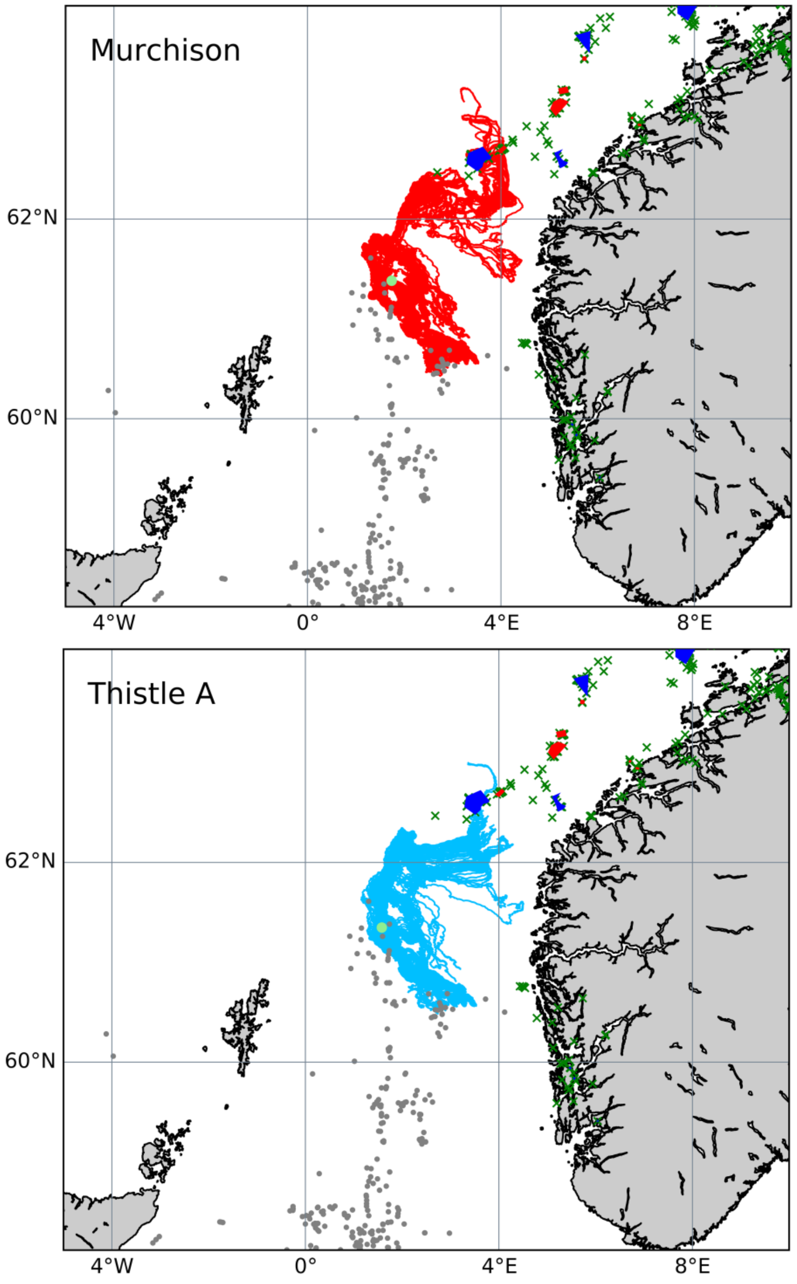

Supplement: Supplementary file 1 — Supplementary Information [file 41598_2018_29575_MOESM1_ESM.pdf]
